# Community-acquired *Klebsiella pneumoniae* pneumonia in ICU: a multicenter retrospective study

**DOI:** 10.1186/s13613-024-01269-3

**Published:** 2024-04-30

**Authors:** Vincent Grosjean, Simon B. Gressens, Tài Pham, Stéphane Gaudry, Hafid Ait-Oufella, Nicolas De Prost, Julien Mayaux, Emmanuel Guerot, Véronique Leflon-Guibout, Noémie Mayer, Frédéric Bert, Nathalie Gault, Clément R. Massonnaud, Damien Roux

**Affiliations:** 1 AP-HP, Service de Médecine Intensive Réanimation, Université Paris Cité, Hôpital Louis Mourier, DMU ESPRIT, 178 Rue des Renouillers, Colombes CEDEX, F-92700 France; 2 Service de Medecine Intensive-Réanimation, Hôpital de Bicêtre, Groupe de recherche CARMAS, AP-HP, DMU CORREVE, FHU SEPSIS, Hôpitaux Universitaires Paris-Saclay, Le Kremlin-Bicêtre, France; 3 Service de Réanimation Médico-Chirurgicale, AP-HP, Hôpital Avicenne, Bobigny, F-93000 France; 4 Service de Médecine Intensive-Réanimation, Hôpital Saint-Antoine, AP-HP, Sorbonne Université, Paris, F-75012 France; 5 AP-HP, Hôpital Henri Mondor, Service de Médecine Intensive Réanimation, Créteil, France; 6 Service de Médecine Intensive-Réanimation, Département R3S, AP-HP, Groupe Hospitalier Universitaire APHP-Sorbonne Université, Site Pitié-Salpêtrière, Paris, F-75013 France; 7 AP-HP, Georges Pompidou European Hospital, Réanimation médicale, Paris, F-75015 France; 8 Laboratoire de Bactériologie, AP-HP, Hôpital Beaujon, Clichy, France; 9 AP-HP, Hôpital Bichat, Département d’Épidémiologie, Biostatistique et Recherche Clinique, Paris, F-75018 France; 10 Université Paris Cité, INSERM, IAME, Paris, F-75018 France; 11 Université Paris Cité, INSERM, CNRS, Institut Necker-Enfants Malades, Paris, F-75015 France; 12 Université Paris-Saclay, UVSQ, Université Paris-Sud, Inserm U1018, Equipe d’Epidémiologie Respiratoire Intégrative, CESP, Villejuif, F-94807 France; 13 Université Paris-Est-Créteil (UPEC), INSERM U955, Team « Viruses, Hepatology, Cancer », Créteil, France

**Keywords:** *Klebsiella pneumoniae*, Community-acquired pneumonia, Bacterial pneumonia, Hypermuscoviscosity, Prognosis, *Streptococcus pneumoniae*, Virulence

## Abstract

**Background:**

Alongside the recent worldwide expansion of hypervirulent *Klebsiella pneumoniae* (KP) infections, the available literature regarding cases of community acquired pneumonias (KP-CAP) remains scarce but reports a strikingly high and early mortality. We performed a retrospective multicenter study (7 ICU in France) between 2015 and 2019, comparing prognosis and severity of KP-CAP versus *Streptococcus pneumoniae -* CAP (SP-CAP).

**Methods:**

For each KP-CAP, three SP-CAP admitted in ICUs within the same center and within the same 6-month window were selected. When available, KP strains were studied, and bacterial virulence was genetically assessed for virulence factors. The primary outcome was in-hospital mortality. Associations between clinical outcomes and type of infection were tested using univariate and multivariate logistic regressions, adjusted for pairing variables.

**Results:**

Twenty-seven KP-CAP and 81 SP-CAP were included. Respective in-hospital mortality rates were 59% (*n* = 16) and 17% (*n* = 14, *p* < 0.001), despite adequate antibiotic therapy. KP-CAP median time from admission to death was 26.9 h [IQR 5.75–44 h] and were significantly associated with higher rates of multiple organ failures (93% vs. 42%, *p* < 0.001), disseminated intravascular coagulation (12% vs. 1.3%, *p* = 0.046), septic shock (median lactate on ICU admission 4.60 vs. 2.90 mmol/L, *p* = 0.030) and kidney failure (KDIGO-3: 87% vs. 44%, *p* < 0.001). Interestingly, alcoholism was the only identified predisposing factor of KP-CAP. Severity on ICU admission (2-fold higher for KP-CAP) was the only factor associated with mortality in a multivariate analysis.

**Conclusion:**

We described a strong association between KP-CAP infection and higher and earlier mortality when compared to SP-CAP. Moreover, alcoholism was the sole predisposing factor associated with KP-CAP infection. These findings should raise awareness of clinicians involved in the management of severe CAP about this microbiological etiology. Future prospective studies are needed to confirm these results and to design strategies to improve the prognosis of such infections.

**Supplementary Information:**

The online version contains supplementary material available at 10.1186/s13613-024-01269-3.

## Background

Community acquired pneumonia (CAP) is a leading cause of death worldwide [1, 2]. CAP mortality rate is particularly high in the intensive care setting (ICU) where it approaches 20%, all microbial causes combined [3, 4]. *Klebsiella pneumoniae* (KP) was first described in the lungs of alcoholic patients dying from severe CAP, known as Friedlander pneumonia [5]. However, KP remains most known in western countries for its classical pathotype responsible for hospital-acquired infections in fragile subjects and concerns due to the acquisition of antibiotic resistance, notably carbapenemases [6]. Another and highly threatening KP pathotype is hypervirulent-KP (HvKP). HvKP has rapidly spread worldwide since its first description in 1986 in Taiwan, with a clinical signature of multiple secondary infectious foci, community-acquired liver abscesses among young, or diabetic, or otherwise healthy patients [7]. This clinical phenotype is associated with hypermucoviscosity on agar plate culture (identified by string test), reflecting overproduction of its external capsule [8]. Mortality from those HvKP infections is usually low and concern arise from their functional outcome due to possible eye and brain infections [9]. 

During HvKP expansion, cases of CAP due to KP (KP-CAP) were reported, with a strikingly high and early mortality, ranging from 55 to 100%, first in Taiwan and later in France [10–15]. In comparison, a recent 1-year survey of all French ICUs found that the 30-day mortality of *Streptococcus pneumoniae* (SP) CAP (which is the most frequent cause CAP in the ICU setting), was about 23% [16]. 

Hence, KP-CAP requiring ICU admission could be an emerging, deadly infection with a possible involvement of HvKP strains and would likely be associated with a higher mortality than of SP-CAP. Former studies on KP-CAP reported high mortality over small data samples and are difficult to compare due to heterogenous setting, patient selection, various epidemiological settings and sometimes lack of control group.

To address these questions, we designed a retrospective multicenter study including ICU patients to evaluate the prognosis of KP infections presenting as CAP, compared to CAP caused by SP (SP-CAP).

## Methods

### Study design and setting

We conducted a retrospective multicenter case-cohort study among 7 ICUs in the Ile-de-France region of France (Paris and suburb hospitals), between January 1st 2015 and, December 31st 2019.

Participating centers were Louis Mourier hospital (Colombes), EOLE ICU (Pitié-Salpêtrière hospital, Paris), Henri Mondor hospital (Creteil), Kremlin Bicêtre hospital (Kremlin-Bicêtre), Avicenne hospital (Bobigny), Saint Antoine hospital (Paris) and Georges Pompidou European Hospital (Paris).

Cases of KP-CAP were paired with controls, SP-CAP, according to center and year of admission (see statistical analysis below). *S. pneumoniae* was chosen as a control micro-organism due to its well described mortality rates [16, 17], as well as its widespread distribution across centers, to limit the risk of center-specific recruitment of other GNR-associated pneumonias.

### Data origin and data collection

Patients were selected by screening the *Programme de Medicalisation des Systèmes d’information (PMSI)* database of each center, centralizing encoded diagnosis based on the International Classification of Diseases, 10th Revision (ICD-10).

Diagnosis were reviewed by the investigators based on electronic medical records by a team of trained clinicians (VG, SBG and DR). Data was anonymized and collected on a Redcap database hosted on the *Assistance Publique – Hôpitaux de Paris* (APHP) servers.

### Study population

All patients with an age over 18 admitted to ICUs with a final diagnosis of KP-CAP or SP-CAP were screened. We included all patients with monobacterial KP-CAP or SP-CAP. Experts confirmed CAP diagnosis by reviewing clinical, biological, microbiological and radiologic data allowing for differential diagnosis exclusion (i.e. cardiogenic pulmonary edema, exacerbation of chronic obstructive pulmonary disease with no evidence of CAP, etc.). We excluded nosocomial pneumonias (defined as pneumonia developed 48 h after hospital admission), health care associated pneumonias (defined as patients hospitalized within 3 months before admission including recurrent stays for specific treatment administration, or patients living in an institution) as well as aspiration pneumonias (defined as pneumonias occurring after an evocative event in the medical story). The initial antibiotic regimen (initiated within the first six hours of ICU admission) was deemed adequate if it aligned with the results of antibiotic susceptibility testing (bacterial isolate susceptible to one or more antibiotic).

### Study objectives and study criteria

Study objectives were first, to assess the prognosis of KP-CAP infection for patients admitted in an intensive care unit and secondly to assess the severity, predisposing factors, and virulence of KP-CAP infection as compared to those of SP-CAP.

Primary outcome was hospital mortality. Secondary outcomes included (1) evaluation of severity (reflected by the following criteria: Simplified acute physiology score II (SAPS II) on ICU admission, highest Sepsis related organ failure assessment (SOFA) score, within 72 h of ICU stay, multiple organ failure occurrence, septic shock rates [18], acute respiratory distress syndrome (ARDS) [19] rates and mechanical ventilation duration, acute kidney injury rates and severity [20]), (2) predisposing factors (alcoholism, diabetes, immunosuppression and other comorbidities) and (3) bacterial virulence (positivity of blood cultures, search for secondary localization with lumbar puncture or imaging).

### Microbiology

When available, KP strains were studied, and bacterial virulence genes were genetically assessed with multiplex PCR testing as described by Compain et al. for the detection of seven virulence factors and K1/K2 capsular serotypes. [21] Standard bacteriological procedures are described in Appendix material 1.

### Statistical analysis

Categorical variables were expressed as percentages, quantitative variables as median [interquartile range, IQR]. Normal distribution of variables was tested by Shapiro-Wilk test, and comparisons of independent quantitative and qualitative variables between groups were performed using the Wilcoxon test and the Kruskal-Wallis non-parametric tests as appropriate.

Associations between the bacterial species responsible for the infection and clinical outcomes were tested using univariate and multivariate logistic regressions, and expressed as odds ratios (OR) and their 95% confidence intervals (CI 95%). Multivariate models were adjusted for pairing variables.

A p value < 0.05 was considered significant. Data were analyzed using R 4.1.2 version (http://www.rproject.org).

#### Pairing

Pairing was achieved by randomly selecting 3 SP-CAP for 1 KP-CAP, admitted in ICUs within the same center and within the same 6-month window (a one-year window was used if not available), to account for caring practices bias.

## Results

We included a total of 108 patients in the final analysis: 27 KP-CAP and 81 SP-CAP. Appendix Fig. 1 describes the flowchart. Their baseline characteristics are displayed in Table 1 and in Appendix Table 1. Briefly, there was no statistical difference between the two groups except alcohol use disorder which was significantly associated with KP-CAP patients (58% vs. 22%, *p* = 0.002). Interestingly, we did not observe significant difference for immunosuppression, cancer, cirrhosis, chronic kidney disease (Table 1), neither for Charlson comorbidity index (median 4 [2, 4] vs. 4 [2, 5]). There was no statistical difference in recruitment between centers.

**Fig. 1:**
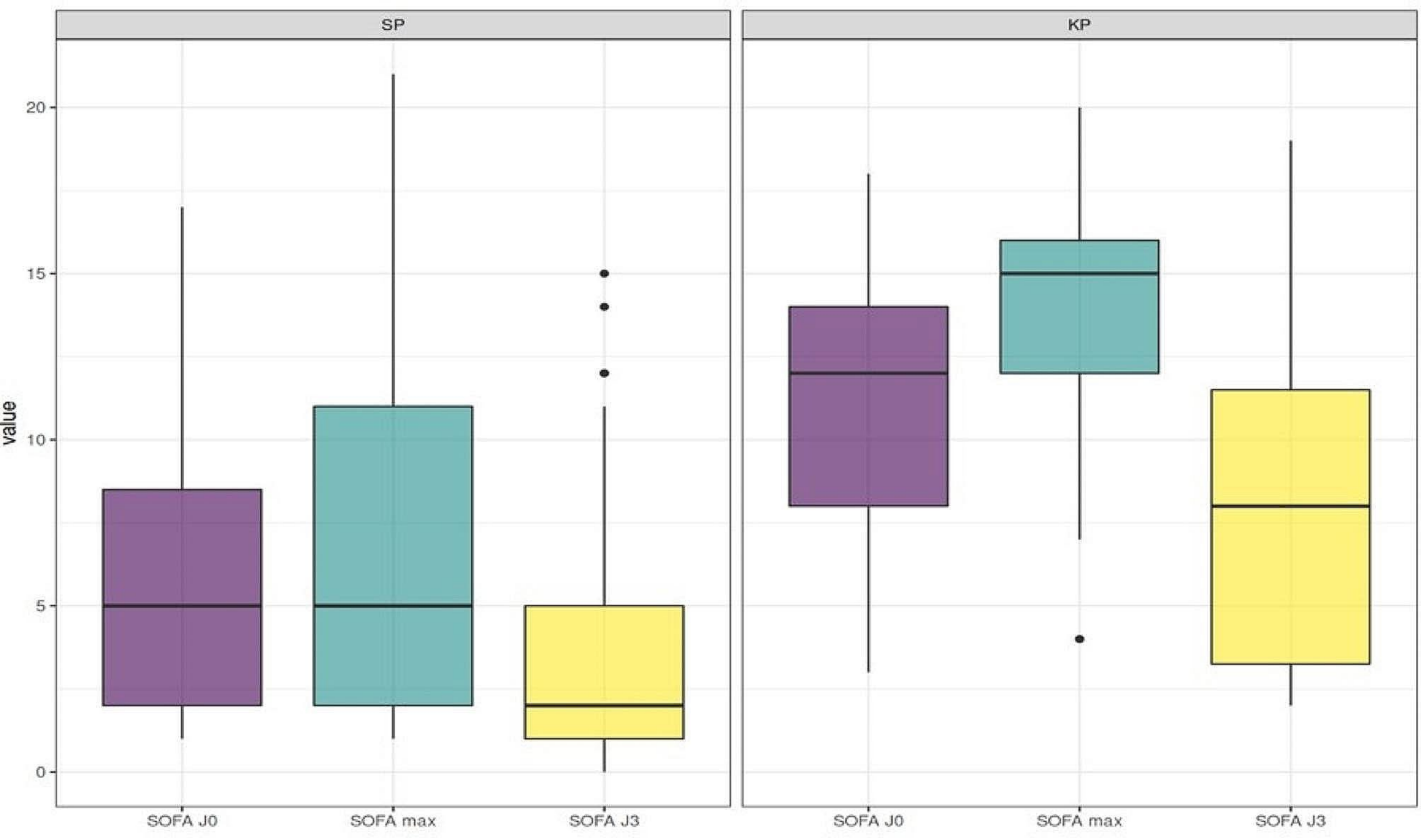
Evolution of SOFA scores from admission to day 3. SOFA scores from admission to day 3 for SP-CAP (left panel) and KP-CAP (right panel) showing boxplots for SOFA at admission (day 0, purple), maximum SOFA from admission to day 3 (blue) and at day 3 (yellow) - SOFA Day 0: KP = 12 (8, 14) vs. SP = 5 (2, 8), *p* < 0.001 - Max SOFA (Day 0-Day 3): KP = 15 (12, 16) vs. SP = 5 (2, 11), *p* < 0.001 - SOFA Day 3: KP = 2 (1, 5) vs. 3 (1, 7), NS

**Table 1:** Characteristics of the population according to the underling infection

	N	KP-CAP, *N* = 27	SP-CAP, *N* = 81	*p*-value
Age (years)	108	68 [56, 80]	73 [61, 81]	0.3
Sex (Male)	108	22 (81%)	53 (65%)	0.12
Body Mass Index (kg/m^2^ )	80	23.8	25.0	0.5
Alcoholism (> 20 g/day)	91	11/19 (58%)	16/72 (22%)	0.002
Charlson s Comorbidity Index (CCI)	108	4 [2, 4]	4 [2, 5]	0.14
Diabetes	106	8/26 (31%)	19/80 (24%)	0.5
Smoking	97	11/22 (50%)	28/75 (37%)	0.3
COPD	106	7/26 (27%)	25/80 (31%)	0.7
Chronic respiratory failure	107	1/27 (3.7%)	1/80 (1.3%)	0.4
Chronic cardiac failure	105	1/26 (3.8%)	9/79 (11%)	0.4
Cirrhosis	105	2 (8.0%)	4 (5.0%)	0.6
Cancer	106	2 (7.7%)	7 (8.8%)	> 0.9
Immunosuppression	107	4 (15%)	15 (19%)	> 0.9
SAPS II (admission)	108	82 [56, 92]	40 [28, 62]	< 0.001
SOFA (admission)	104	12 [8, 14]	5 [2, 8]	< 0.001

*KP-CAP Klebsiella pneumoniae* community-acquired pneumonia; *SP-CAP Streptococcus pneumoniae* community-acquired pneumonia; *SAPS II* Simplified Acute Physiology Score II; *SOFA* Sepsis related organ failure assessment. Data is n (%) or median [IQR]

In-hospital mortality was higher in the KP-CAP group as compared to the SP-CAP group (59% vs. 17% respectively, *p* < 0.001). The peak of mortality occurred during the first days following admission, as suggested by a median delay from admission to death among KP-CAP patients of 26.9 h [5.75–44 h].

In an univariate logistic regression, KP-CAP was associated with in-hospital mortality (OR: 6.86, 95% CI: 2.67–18.5, *p* < 0,001), which was confirmed in a multivariate analysis taking into account the center of recruitment and the year of admission (OR 7.85, 95% CI: 2.92–22.7, *p* < 0.001). When adjusting on SAPSII score on admission, the association between KP-CAP and hospital mortality was no longer significant (OR 1.84, 95% CI: 0.43–7.61, *p* = 0.4) (Appendix Table 2). Of note, median SAPSII score on admission was higher among KP-CAP versus SP-CAP patients (82 [56, 92] vs. 40 [28, 62] respectively, *p* < 0.001) (Table 1). SOFA score on admission was also significantly higher among KP-CAP patients (12 [8, 14]) than that of SP-CAP (5 [2, 8], *p* < 0.001), as was the maximum SOFA score between ICU admission and day 3 (15 [12, 16] vs. 5 [2, 11] respectively, *p* < 0.001) (Table 1; Fig. 1). To be noted, time from first symptoms to ICU admission were available for 17/27 KP-CAP patients (median of 48 h, IQR [24–96]) and for 65/81 SP-CAP cases (median 72 h, IQR [48–120]), which was not significantly different (*p* = 0.9).

During ICU stay, KP-CAP patients presented significantly more severe phenotypes with higher rates of multiple organ failures (93% vs. 42%, *p* < 0.001), disseminated intravascular coagulation (12% vs. 1.3%, *p* = 0.046), septic shock (median lactate on ICU admission 4.60 vs. 2.90 mmol/L, *p* = 0.030) and kidney failure (KDIGO-3: 87% vs. 44%, *p* < 0.001). There was no difference in ARDS (78% vs. 79%, *p* > 0.9) even though KP-CAP were more susceptible to receive endotracheal intubation for mechanical ventilation (85% vs. 42%, *p* < 0.001). (Table 2).

**Table 2:** Prognosis and severity outcomes according to the pathogen

	N	KP-CAP, *N* = 27	SP-CAP, *N* = 81	*p*-value
**Primary outcome**				
In-hospital mortality	108	16/27 (59%)	14/81 (17%)	< 0.001
Limitations of life-sustaining therapy (among deceased)	30	5/16 (31%)	8/14 (57%)	0.3
**Secondary outcomes**				
Multiple organ failure	107	25/27 (93%)	34/81 (42%)	< 0.001
Disseminated intravascular coagulation	106	3/26 (12%)	1/80 (1.3%)	0.046
**Septic Shock**				
Lactate on ICU admission	52	4.60 [3.50, 7.00]	2.90 [2.38, 5.05]	0.030
Norepinephrine use	107	21/27 (78%)	28/80 (35%)	< 0.001
**Respiratory failure**				
Endotracheal Intubation and mechanical ventilation	108	23/27 (85%)	34/81 (42%)	< 0.001
ARDS	57	18/23 (78%)	26/34 (76%)	> 0.9
**Kidney failure**				
KDIGO 3 renal failure	73	20/23 (87%)	22/50 (44%)	< 0.001
Persistent renal failure at day 2	60	13/14 (93%)	22/46 (48%)	0.003
**Microbiology**				
Viral coinfections	108	2 (7%)	17 (21%)	0.14

*KP-CAP Klebsiella pneumoniae* community-acquired pneumonia; *SP-CAP Streptococcus pneumoniae* community-acquired pneumonia; *ARDS* acute respiratory distress syndrome; *KDIGO* kidney disease: improving global outcomes. Data is n/N (%) or median [IQR]

Underlying comorbidities were not solely driving severity: correlation analysis through linear regression showed significantly higher SAPS on admission among KP-CAP patients when adjusted for Charlson Comorbidity Index (mean difference 27 points (95%CI 17–38, *p* < 0.001) (Appendix Fig. 2).

Moreover, antibiotic susceptibility testing showed that the vast majority of initial empiric antibiotic therapy was adequate as only one was inadequate (a 75-year-old woman diagnosed with KP-CAP and influenza virus co-infection, with KP expressing extended spectrum beta-lactamase, revealing an agammaglobulinemia, who eventually survived). KP-CAP patients presented more positive blood cultures as compared to SP-CAP patients, although this was not significant (52% vs. 23%, *p* = 0.14) (Table 3). Initial antibiotic regimens are available in Appendix Table 3. No early change of antibiotics (within 24 h of the first line) was noted in the cohort. Prevalence of co-infection with respiratory viruses were not statistically different in KP-CAP (2/27, both influenza virus) and SP-CAP (17/81, details in Appendix Table 4, *p* = 0.14).

**Table 3:** Microbiological diagnosis and antibiotic therapy in the study population

	N	KP-CAP, *N* = 27	SP-CAP *N* = 81	*p*-value
**Positive diagnostic testing**				
Blood culture	42	14 (52%)	29 (23%)	0.14
Sputum	22	6 (22%)	16 (13%)	0.78
Tracheal aspiration	29	15 (55%)	14 (15%)	< 0.001
Protected distal aspiration	6	1 (3.7%)	5 (11%)	
Bronchoalveolar lavage	12	6 (22%)	6 (5%)	0.03
Antigenuria	53	ND	53 (43%)	
**Total**	164	42	122	
**Antibiotic therapy**				
Inadequacy of initial antibiotic prescription.		1	0	
Resistance to antibiotics		1 Extended spectrum beta-lactamase	3 *S. pneumoniae* with low susceptibility or resistance to penicillin (MIC* > 0.125 mg/L)	

*KP-CAP Klebsiella pneumoniae* community acquired pneumonia; *SP-CAP Streptococcus pneumoniae* community acquired pneumonia; *MIC* minimum inhibitory concentration; *ND* not determined

It is noteworthy that, when performed within the first 48 h (11/27), CT scans only showed signs of classical CAP (lobar consolidation = 9, interstitial infiltrate = 2, pleurisis = 1) without specific pattern associated with KP-CAP. In addition, when performed in KP-CAP patients, cerebrospinal fluid analysis (*N* = 4) was normal and abdominal CT scan did not show hepatic abscess (*N* = 9).

Finally, available KP strains (*n* = 4) were screened retrospectively for hypervirulent genotype by multiplex PCR as an exploratory analysis (Appendix Table 5). All tested isolates were compatible with hypervirulent strains as they expressed either a K2 or a K1 serotype (2 each) and were positive for classical Hv-KP virulence factors i.e., hypermucoviscosity (regulator of mucoid phenotype A *(rmpA)*, all positives), and siderophores (*iutA, entB, ybts*, all positives*).*

## Discussion

Our study showed that infection with monobacterial KP-CAP was associated with a high in-hospital mortality, strikingly superior to that of SP-CAP (58% vs. 17%, *p* < 0.001) in an ICU setting.

These mortality rates are in accordance with previous studies focusing on KP-CAP. Rafat et al. and Moutel et al. reported mortality rates of 50% and 56%, respectively, whereas others reported even higher rates (75% in Paganin et al. or 100% in Jong et al.) [10, 11, 14, 15]. SP-CAP mortality observed in the present study is close to the 30-day mortality of 23% observed in a recent ICU study in France [16]. To our knowledge, this is the first retrospective cohort describing with detailed clinical and microbiological data, the prognosis and severity of KP-CAP infection in an ICU setting with a robust comparator, SP-CAP, the first and most well-described cause of CAP.

Interestingly, SAPS-II and SOFA scores on ICU admission captured the earlier severity of KP-CAP presentation compared to SP-CAP. These scores captured both the clinical severity and the medical history burden of the patients. [22, 23] This initial dramatic presentation might have been the principal driving factor of the observed mortality, as association between mortality and bacterial cause of the CAP was no longer significant when adjusted of SAPS-II score on admission. This clinical severity continued to worsen rapidly during the first 72 h after admission among KP-CAP patients despite adequate antibiotic therapy. Notably, one patient received a combination of beta-lactam/beta-lactam inhibitor as a first line regimen. This patient died within the first 24 h after ICU admission, and it cannot be excluded that this antibiotic regimen was suboptimal due to PK/PD issues and a potential marked inoculum effect even though the bacterial isolate was deemed susceptible. Even though respiratory distress severity was similar to that of SP-CAP as previously reported, KP-CAP patients displayed a more systemic disease with significantly more multiple organ failure, septic shock, kidney failure, and disseminated intravascular coagulation. Such clinical course resulted in premature death as the median time from admission to death was 27 h [IQR 5.75–44 h], a quite unusual figure in ICU patients hospitalized for lung infections.

Age and comorbidities were equally distributed between KP and SP-CAP groups, including classically described factors associated with CAP mortality. [24–27] Alcohol misusage was the only risk factor significantly more prevalent among KP-CAP patients, confirming previous data [10–14]. Indeed, correlation analysis between Charlson Comorbidity Index (CCI) and SAPS II on ICU admission revealed a significantly superior severity score in KP compared to SP-CAP patients (average + 27 SAPS II points for every CCI point-increment, Appendix Fig. 2). Therefore, considering roughly similar baseline characteristics between SP and KP-CAP patients, one could hypothesize that the observed increased mortality among KP-CAP was mainly driven by sepsis severity and *K. pneumoniae* pathogenesis itself. Moreover, when available, pulmonary and abdominal CT scan analysis failed to reveal a specific pattern for KP-CAP. Because of the retrospective nature of the study, biological markers were not collected as there were considerable differences in practices across the different centers, preventing efficient comparisons. However, this analysis is hampered by the lack of systematic assessment of biomarkers and radiological evaluation in all participating centers.

Exploratory analysis revealed that genomes of all available KP strains (*n* = 4) carried hypervirulence genes typically associated with Hv-KP, both during CAP and liver abscesses. [9, 13, 14, 21] Of those four cases, two died rapidly after admission (75 year-old woman: 31 h, 67 year-old man: 23 h), one died 4 months after the recorded episode (75-year-old man) and one survived (64 year-old man presenting with subacute excavated pneumonia and pleurisies).

Our study has limitations. First, as the identification of patients through French ICD-PMSI database might have induced bias in patient recruitment, [28] we chose strict inclusion criteria to maximize uniformity and comparability in our population, limiting our sample size. Second, delay from hospital admission to antibiotic therapy, a known risk factor for poor prognosis, was not timed precisely in the charts even though the timeframe of initial antibiotic introduction could be restricted to the first six hours based on the electronic records available. However, considering the initial presentation of KP-CAP and frequency of septic shock, it would seem unlikely that antibiotic therapy would have been delayed as compared to SP-CAP patients. Third, the majority of KP strains in our study was not available for genomic analysis. This drawback could reflect the relative unfamiliarity with this potential HvKP presentation or a selection bias in strains storage, particular strains (for example with a hypermucoviscous phenotype) being more likely stored. Our study hints for a broader education of clinicians and microbiologists involved in the care of these patients as similar presentations (i.e., severe septic shock with multiple organ failure and early mortality) could reveal a higher prevalence of hypervirulent strains in respiratory presentations than previously evaluated.

In conclusion, we showed that in comparison to SP-CAP, monobacterial infection with non-health care related KP-CAP was associated with significantly more severe presentation on ICU admission and worse prognosis, including rapidly occurring death.

Our study prompts for future broader prospective studies and registries aiming to decipher the epidemiology and virulence mechanisms underlying such severe conditions. Previous rapid dissemination of HvKP strains (i.e. in Taïwan [7, 8, 29]) as well as recent outbreaks of carbapenem resistant HvKP strains highlight the urgent need of close surveillance of these lethal infections. [30–32].

### Electronic supplementary material

Below is the link to the electronic supplementary material.

Supplementary Material 1

Supplementary Material 2

## Data Availability

The datasets used and/or analyzed during the current study are available from the corresponding author on reasonable request.
