# Supplementary material for: Community-acquired Klebsiella pneumoniae pneumonia in ICU: a multicenter retrospective study
Source: Ann Intensive Care. 2024 Apr 30;14:69. doi: 10.1186/s13613-024-01269-3 (PMC11061059; doi:10.1186/s13613-024-01269-3)
Supplement: Supplementary file 2 — Supplementary Material 2 [file 13613_2024_1269_MOESM2_ESM.docx]

**Community-acquired *Klebsiella pneumoniae* pneumonia in ICU: a multicenter retrospective study**

**Additional file**

**Appendix methodology for standard bacteriological procedures**

Respiratory sample cultures were performed by inoculation agar plates and incubated for 72h under aerobic and anaerobic atmosphere (blood cultures were incubated for 7-10 days). Identification of bacterial species was performed by matrix-assisted laser desorption/ionization time-of-flight mass spectrometry (MALDI-TOF/MS, Microflex; Bruker Daltonics, Bremen, Germany) on colonies.  Antimicrobial susceptibility testing was performed independently in each center using the disk diffusion method according to the EUCAST guidelines. Urinary antigen testing was performed as recommended by the manufacturer.

**Appendix Table 1:** extended baseline characteristics of the study population

|  | **N** | **KP-CAP,**  **N = 27** | **SP-CAP,**  **N= 81** | **p-value** |
| --- | --- | --- | --- | --- |
| **Age (Years)** | 108 | 68 (56, 80) | 73 (61, 81) | 0.3 |
| **Sex (Male)** | 108 | 22 (81%) | 53 (65%) | 0.12 |
| **Body Mass Index (kg/m^2^)** | 80 | 23.8 (22.0, 28.9) | 25.0 (22.2, 29.8) | 0.5 |
| **Alcoholism (> 20g/day)** | 91 | 11 (58%) | 16 (22%) | 0.002 |
| **Diabetes** | 106 | 8 (31%) | 19 (24%) | 0.5 |
| **Diabetes type 2** |  | 8 (100%) | 18 (95%) |  |
| **Controlled diabetes** | 14 | 2 (50%) | 7 (70%) | 0.6 |
| **Multiple complications** | 19 | 2 (40%) | 8 (57%) | 0.6 |
| **Smoking** | 97 | 11 (50%) | 28 (37%) | 0.3 |
| **COPD** | 106 | 7 (27%) | 25 (31%) | 0.7 |
| **Chronic respiratory failure** | 107 | 1 (3.7%) | 1 (1.2%) | 0.4 |
| **High blood pressure** | 106 | 12 (48%) | 41 (51%) | 0.8 |
| **Chronic heart failure** | 105 | 1 (3.8%) | 9 (11%) | 0.4 |
| **Chronic kidney failure** | 104 | 0 | 4 (5.0%) | 0.6 |
| **Stage (CKD)** | 3 |  |  | >0.9 |
| **3** |  | / | 2 (67%) |  |
| **4** |  | / | 1 (33%) |  |
| **Cirrhosis** | 105 | 2 (8.0%) | 4 (5.0%) | 0.6 |
| **Cancer** | 106 | 2 (7.7%) | 7 (8.8%) | >0.9 |
| **Stage** | 8 |  |  | >0.9 |
| **Remission** |  | 2 (100%) | 3 (50%) |  |
| **Localized** |  | 0 (0%) | 1 (17%) |  |
| **Mestastatic** |  | 0 (0%) | 2 (33%) |  |
| **Immunosuppression** | 107 | 4 (15%) | 15 (19%) | >0.9 |
| **Systemic steroïds** |  |  | 1 |  |
| **Cancer chemotherapy** |  |  | 3 |  |
| **Indolent hemopathy** |  |  | 2 |  |
| **Malignant hemopathy** |  | 1 | 3 |  |
| HIV, controled |  |  | 3 |  |
| HIV not controled |  | 2 | 2 |  |
| Others |  | 1 (Agammaglobulinemia) | 1 (Azathioprine) |  |
| Dementia | 106 | 0 (0%) | 2 (2.5%) | / |
| Dysphagia | 108 | 0 | 0 | / |
|  |  |  |  |  |
| Charlson comorbidity index | 108 | 4 (2, 4) | 4 (2, 5) | 0.14 |

*KP-CAP Klebsiella pneumoniae* community acquired pneumonia; *SP-CAP Streptococcus pneumoniae* community acquired pneumonia; *SAPS II* Simplified Acute Physiology Score II; *SOFA* Sepsis related organ failure assessment; *COPD* chronic obstructive pulmonary disease; *CKD* chronic kidney disease.

**Appendix Table 2:** univariate and multivariate logistic regression for in-hospital mortality of patients in this study.

| **Variable** | **Univariate analysis** | | | **Multivariate analysis – model 1** | | | **Multivariate analysis – model 2** | | |
| --- | --- | --- | --- | --- | --- | --- | --- | --- | --- |
|  | **OR** | **95%CI** | **p-value** | **OR** | **95%CI** | **p-value** | **OR** | **95%CI** | **p-value** |
| **KP-CAP** | 6.86 | 2.67 – 18.5 | <0.001 | 7.85 | 2.92 - 22.7 | <0.001 | 1.84 | 0.43, 7.61 | 0.4 |
| **SAPS II** | 1.09 | 1.06 – 1.13 | <0.001 | — | — | — | 1.10 | 1.06, 1.15 | <0.001 |
| **Year** | 0.93 | 0.68 – 1.27 | 0.7 | 0.95 | 0.64 – 1.41 | 0.8 | 1.33 | 0.74, 2.56 | 0.4 |
| **Center** |  |  |  |  |  |  |  |  |  |
| LMR | — | — | — | — | — | — | — | — | — |
| AVI | 1.50 | 0.37, 5.95 | 0.6 | 1.61 | 0.34, 7.54 | 0.5 | 0.87 | 0.10, 7.27 | 0.9 |
| KB | 1.24 | 0.36, 4.29 | 0.7 | 1.37 | 0.33, 5.89 | 0.7 | 0.69 | 0.09, 5.29 | 0.7 |
| SAT | 0.60 | 0.08, 3.05 | 0.6 | 0.58 | 0.06, 3.75 | 0.6 | 1.40 | 0.08, 18.1 | 0.8 |
| HEGP | 3.00 | 0.73, 12.9 | 0.13 | 3.72 | 0.78, 18.8 | 0.10 | 2.08 | 0.24, 19.6 | 0.5 |
| EOLE | 0.43 | 0.02, 3.07 | 0.5 | 0.35 | 0.02, 3.15 | 0.4 | 0.51 | 0.01, 13.0 | 0.7 |
| HMR | 1.00 | 0.13, 5.62 | >0.9 | 1.01 | 0.11, 6.94 | >0.9 | 0.35 | 0.01, 8.91 | 0.5 |

KP-CAP was associated to hospital death with an OR of 6.86, (95% CI: 2.67- 18.5, p<0,001). Taking in account center of recruitment and year of admission, KP-CAP infection OR for in hospital death was 7.85, (95% CI: 2.92- 22.7, p<0,001). This was no longer significative after adjusting by SAPSII on ICU admission (OR 1.84, 95% CI 0.43-7.61, p = 0.4).

**Appendix Table 3**: First line antibiotic regimen and associated mortality

|  | **3GC/4GC** | **Penicillin** | **Betalactam (BL) +**  **BL inhibitor** | **Aminoglycoside** | **Quinolone^1^** | **Anti-MRSA** | **Macrolide** | **Other antibiotic^2^** |
| --- | --- | --- | --- | --- | --- | --- | --- | --- |
| **Total % (n)** | 81.5% (89/108) | 6.5% (7/108) | 10.2% (11/108) | 6.5% (7/108) | 20.4% (22/108) | 0.9% (1/108) | 53.7% (58/108) | 8.33% (9/108) |
| **KP % (n)** | 92.6% (25/27) | 0% (0/27) | 7.4% (2/27) | 18.5% (5/27) | 22.2% (6/27) | 3.7% (1/27) | 63% (17/27) | 7.4% (2/27) |
| **SP % (n)** | 77.8% (63/81) | 8.6% (7/81) | 11.1% (9/81) | 2.5% (2/81) | 19.8% (16/81) | 0% (0/81) | 50.6% (41/81) | 8.6% (7/81) |
| *Deceased* | 26/108  (15 KP) | 2/108 (0 KP) | 3/108 (2 KP) |  |  |  |  |  |

In grey: backbone molecule of the antibiotic regimen. Other antibiotics are associated to the beta-lactam (white background) for most patients.

*^1^Only 1 patient did not receive any beta-lactam as first line therapy (received levofloxacin)*

*^2^including Cotrimoxazole (n=5), Rifampicin (n=1), Metronidazole (n=3)*

**Appendix Table 4.** Viral co-infections

| **Identified viruses** | **N** | **KP-CAP, N = 27** | **SP-CAP, N = 81** | **p** |
| --- | --- | --- | --- | --- |
| **Influenza virus** | 7 | 2 (100%) | 5 (29%) |  |
| **Rhinovirus / Enterovirus** | 5 | - | 5 (29%) |  |
| **RSV** | 3 |  | 3 (18%) |  |
| **Coronavirus** | 2 |  | 2 (12%) |  |
| **Human Parainfluenza virus** | 2 |  | 2 (12%) |  |
| **Total** | 19 | 2 (7%) | 17 (21%) | 0.14 |

RSV : Respiratory syncytial virus. Viral co-infections were found in 7% of KP-CAP and 21% of SP-CAP infected patients. Most prevalent virus were Influenza and Picornaviridae (Rhinovirus/enterovirus). There was no statistically significant difference in the proportion of co-infection rates between KP-CAP and SP-CAP patients.

**Appendix Table 5.** Multiplex PCR testing for hypervirulent genotype for all available strains of *Klebsiella pneumoniae*.

|  | **Genes, function and amplicon size (bp*)** | | | | | | | | |
| --- | --- | --- | --- | --- | --- | --- | --- | --- | --- |
| **Strain** | **magA / K1**  Capsular serotype K1 and hypermucoviscosity phenotype | **K2**  Capsular serotype K1 and hypermucoviscosity phenotype | **rmpA**  Regulator of mucoide phenotype A | **iutA**  Siderophore | **entB**  Siderop hore | **Ybts**  Siderophore | **Kfu**  Iron transport and phosphotransferase function | **allS**  allatoin metabolism | **mrkD**  Adhesin type III fimbriae |
| **1** | neg | pos | pos | pos | pos | Pos | neg | neg | pos |
| **2** | pos | neg | pos | pos | pos | pos | neg | pos | pos |
| **3** | neg | pos | pos | pos | pos | pos | neg | neg | pos |
| **4** | pos | neg | pos | pos | pos | pos | pos | pos | pos |

Multiplex PCR testing for hypervirulent genotype for the four available strains of *Klebsiella pneumoniae* of the study population.

First line contains genes tested with the PCR. Lines 2 to 5 are tested strains (1 to 4).

**Appendix Figure 1.** Flowchart of inclusions

CAP: community-acquired pneumonia; ICU: intensive care unit

*A larger pilot screening was done in one of the centers, using more ICD-10 codes (J960: acute respiratory distress, with at least one of the following codes: J150, J156: Pneumoniae due to other Gram-negative bacteria, B961: *K.pneumoniae* as the cause of diseases classified elsewhere). This pilot screening resulted in 41 more screened patients (total of 62 cases), but no additional case was included when compared to only the J960 + J150 combination. Therefore, we used J13/J150 to screen the other centers (n=1003 cases screened).

**Appendix Figure 2:** Relationship between severity at admission and burden of comorbidities.


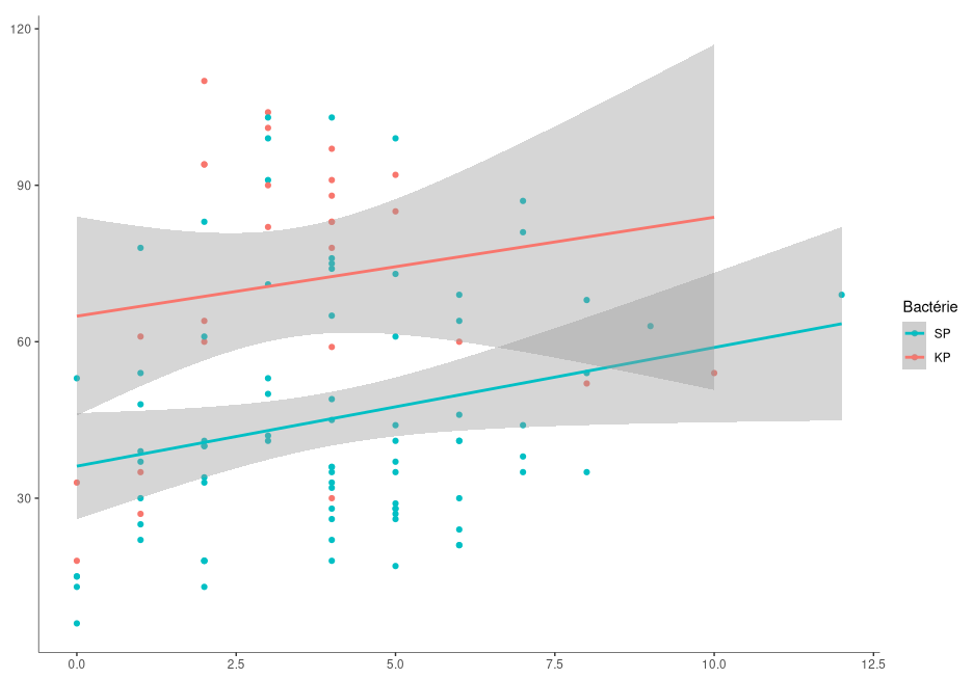


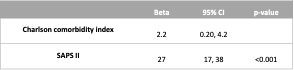


Using linear regression, correlation between Charlson Comorbidity Index (x-axis) and admission SAPSII (y-axis) is represented for both KP-CAP (red line) and SP-CAP (blue line).
